# Serum anti-phospholipase A2 receptor antibody in pathological diagnosis of type 2 diabetes mellitus patients with proteinuria

**DOI:** 10.1038/s41598-023-43766-8

**Published:** 2023-10-03

**Authors:** Huanhuan Zhang, Yuanjie Zhu, Zhijuan Hu, Qiong Liu

**Affiliations:** Department of Nephrology, Hebei General Hospital, Shijiazhuang, 050000 China

**Keywords:** Kidney diseases, Diagnostic markers

## Abstract

Patients with diabetes mellitus complicated with proteinuria can be diabetic nephropathy (DN), diabetic complicated with non-diabetic kidney disease (NDKD), or DN with NDKD. Among these membranous nephropathy accounted for a large proportion of DN with NDRD. At present, serum anti-phospholipase A2 receptor (PLA2R) antibody is widely used in the diagnosis and evaluation of therapy in idiopathic membranous nephropathy, our study aimed to investigate the diagnostic significance of anti-PLA2R antibody in type 2 diabetes mellitus (T2DM) patients with proteinuria, providing a method for patients with contraindications of kidney biopsy. Eighty-seven T2DM patients with proteinuria who went on kidney biopsy were divided into the DN group, idiopathic membranous nephropathy (IMN) group, and others group according to their pathological results. In our study, 52.87% and 28.74% of patients were found to have IMN and diabetic nephropathy respectively. The levels of anti-PLA2R antibody, total cholesterol, triglyceride, and estimated glomerular filtration rate (eGFR) were higher in the IMN group, while the prevalence of diabetic retinopathy (DR), systolic blood pressure (SBP) and HbA1c were higher in the DN group. For T2DM patients with proteinuria, anti-PLA2R antibody (AUC = 0.904, 95%CI 0.838–0.970) has a high diagnostic value for IMN. The duration of diabetes (*OR* = 0.798, *P* = 0.030), eGFR level (*OR* = 1.030, *P* = 0.024), and positive anti-PLA2R antibody (*OR* = 72.727, *P* < 0.001) favor the diagnosis of IMN, while DR (*OR* = 50.234, *P* < 0.001), SBP (*OR* = 1.041, *P* = 0.030), and negative anti-PLA2R antibody (*OR* = 0.008, *P*  = 0.001) is beneficial to the diagnosis of DN. Our study found that NDKD is not uncommon in patients with T2DM and proteinuria, and IMN was the main pathological type. Positive anti-PLA2R antibody has a strong accuracy in the diagnosis of IMN in patients with T2DM and proteinuria.

## Introduction

Type 2 diabetes mellitus (T2DM) has become one of the biggest challenges of global public health in the twenty-first century. Diabetic nephropathy (DN) is a common microvascular complication of T2DM, accounting for about 30–40% of T2DM^1^. DN, a microvascular disease, is glomerular sclerosis caused by abnormal metabolism of diabetes. About one-third of diabetic patients with proteinuria are eventually diagnosed with non-diabetic kidney disease (NDKD)^2^. Research shows that many newly diagnosed DKD patients are NDKD or DN combined with NDKD^3^. In Asia and Europe, the most common NDKD pathological type is idiopathic membranous nephropathy (IMN), while focal segmental glomerulosclerosis (FSGS) is the main pathological type in North America^4–6^. In China, IMN is the most common pathological type in NDKD patients^7, 8^, followed by IgA nephropathy (IgAN)^9^. Moreover, IgAN is also one of the common types in DN with NDKD^9^. In general, DN has a poor prognosis and is one of the common causes of death in diabetic patients. In contrast, NDKD is treatable in most cases. Therefore, it is important to clarify the pathological pattern of diabetic patients with proteinuria for prognosis.

In clinical practice, the diagnosis of DN mainly depends on medical history, clinical manifestations, and laboratory indicators, including diabetic retinopathy (DR), the duration of the disease, the levels of glycosylated hemoglobin (HbA1C), and so on^10, 11^. However, a great part of NDKD is easy to be misdiagnosed. As the gold standard method to diagnose diabetic nephropathy, kidney biopsy is an invasive examination. Complications such as hemorrhage and infection may limit its application. Therefore, a simple and reliable non-invasive laboratory index is needed to detect NDKD patients.

In 2009, Beck detected an antigen normally expressed on the podocyte cell, the M-type phospholipase A2 receptor (PLA2R) by using western blotting^12^, and the PLA2R antibody may cause IMN in up to 80% of patients^13^. Anti-PLA2R antibody is a type I transmembrane receptor and a member of the mammalian mannose receptor family. Anti-PLA2R antibody and PLA2R form an antigen/antibody complex that is deposited on the glomerular basement membrane, activate the complement system, causes damage to podocytes and the glomerular filtration barrier, and eventually results in proteinuria, which offers clinicians and scientists a powerful tool to differentiate primary from secondary forms and monitor disease activity and response to therapy^14^. The anti-PLA2R antibody has a high specificity in comparing IMN with other glomerular diseases. Zhang showed that the positive rate of anti-PLA2R antibody was 33% in 36 patients with tumor-associated membranous nephropathy. However, due to the possibility of the coexistence of membranous nephropathy and malignant tumors, it is difficult to distinguish IMN with malignant tumors from tumor-associated membranous nephropathy^15^. It still can not be called reliable evidence in distinguishing tumor-associated membranous nephropathy but performing routine screening for anti-PLA2R antibody in patients with MN and malignancy is reasonable^16^. However, this kind of error can be reduced by medical history and laboratory examination. In addition, IMN can be treated with cyclophosphamide and corticosteroids, immunosuppressive therapy, rituximab, or supportive therapy^17^. It was demonstrated that reductions in anti-PLA2R antibody titers predicted disease remission regardless of the method used^18, 19^. The increase in antibody titer indicates the development of the disease^20^.

However, there are limited studies on serum anti-PLA2R antibodies in diabetic patients with proteinuria, and the diagnostic effect of anti-PLA2R antibody in this population is still unclear. This study aimed to find the expression of serum anti-PLA2R antibody in patients with T2DM proteinuria and to explore the diagnostic value of antibodies for IMN in this population.

## Materials and methods

### Study population

**Figure 1:**
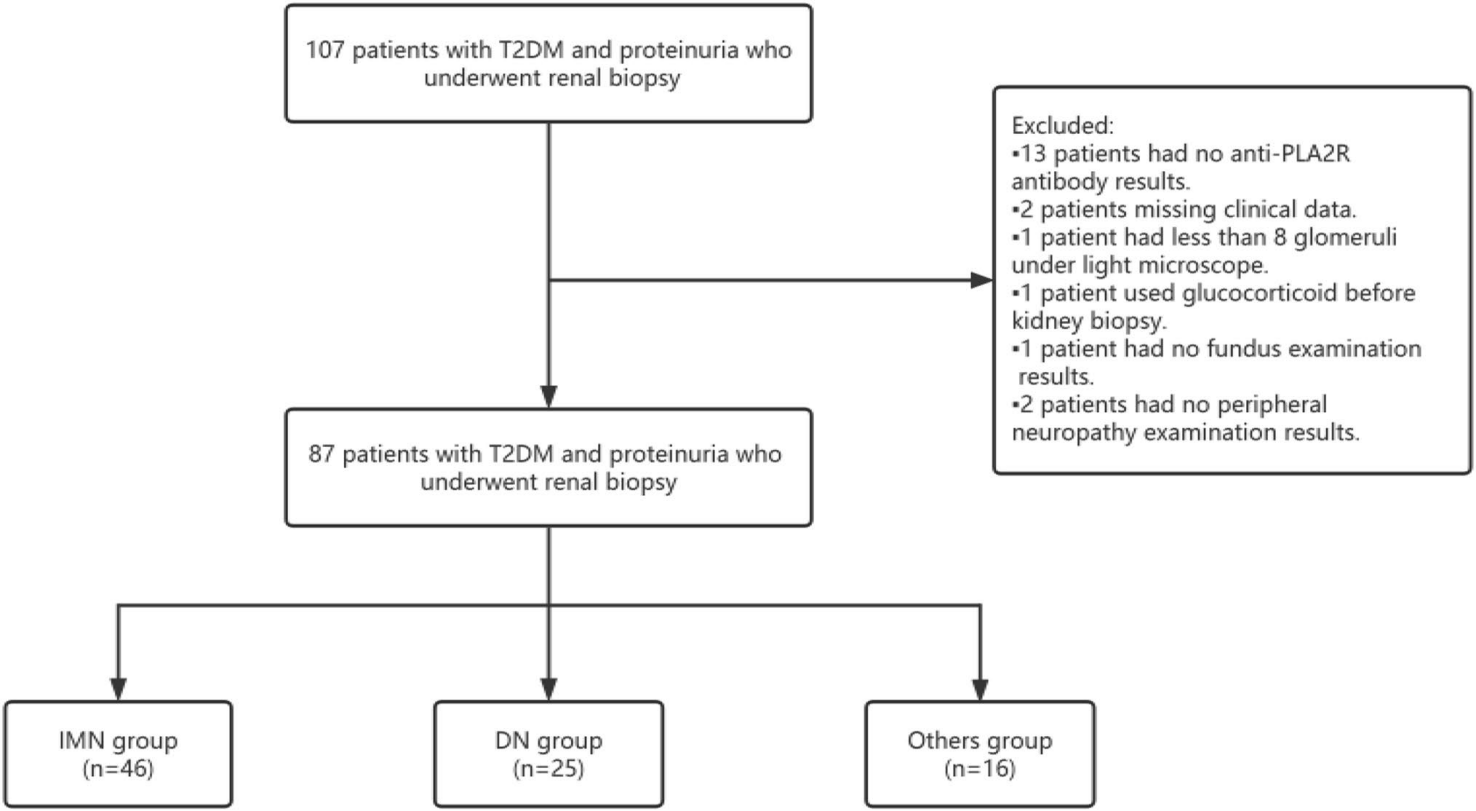
Flow diagram of the study participants.

A total of 107 T2DM patients with 24-h urine protein > 0.5g/d who went on kidney biopsy were recruited in Hebei General Hospital from January 2017 to December 2019. Twenty patients were excluded because of lack of sufficient clinical data, type 1 diabetes, use of steroids, immunosuppressants before kidney biopsy, no anti-PLA2R antibody result, no fundus examination, and no peripheral neuropathy. The remaining 87 patients were divided into the IMN group (n = 46), DN group (n = 25), and T2DM with other glomerulopathy group (n = 16) according to kidney pathological patterns. DN was diagnosed according to the pathological diagnostic criteria established by Tervaert^21^. Obtain informed consent from all recruited patients (Fig. 1). The study was approved by the Institutional Ethics Committee of Hebei General Hospital. The approval number is NO.2021259. We confirm that all analyses were performed according to relevant guidelines and regulations. And confirm the informed consent of all participants.

### Clinical and biochemical assessment:

Age, sex, body mass index (BMI), duration of T2DM, family history of diabetes, hypertension, diabetic retinopathy (DR), diabetic peripheral neuropathy (DPN), systolic blood pressure (SBP), and diastolic blood pressure (DBP) of all patients were collected. Serum albumin (ALB), urea nitrogen (BUN), serum creatinine (Scr), uric acid (UA), triglyceride (TG), total cholesterol (TC), fasting plasma glucose (FPG), twenty-four hours urinary protein (UTP) was measured by Beckman AU5821 automatic analyzer (Beckman Coulter, Miami, FL, USA). HbA1c was determined by high-performance liquid chromatography with Arkray HA-8180 automatic glycated hemoglobin analyzer (ARKRAY, Inc, Kyoto, Japan). The CKD-EPI formula was used to calculate the estimated glomerular filtration rate (eGFR)^22^. Serum anti-PLA2R antibodies levels were measured by using human ELISA kits (Oumeng, Lubeck, Germany). The results were considered positive at a level of ≥ 14RU/ml.

### Statistical analysis

All data were analyzed by using IBM SPSS Statistics software version 25.0. Quantitative variables with normal distribution were expressed as $\bar{\chi}\pm \text{s}$, with abnormally distributed data were expressed as [*M(P25, P75)*]. Categorical variables were expressed as percentages. Multiple groups of quantitative variables were compared using ANOVA or Kruskal-WallisH test. Two group differences in quantitative variables were analyzed using the independent samples *t*-test or Mann–Whitney U test. The Chi-square test was used to compare categorical variables. The receiver operating characteristics (ROC) curve was used to analyze the predictive value of serum anti-PLA2R antibody for IMN. Multivariate logistic regression analysis was performed to identify risk factors of IMN and DN. *P* value < 0.05 was set to statistically significant.

## Results

**Figure 2:**
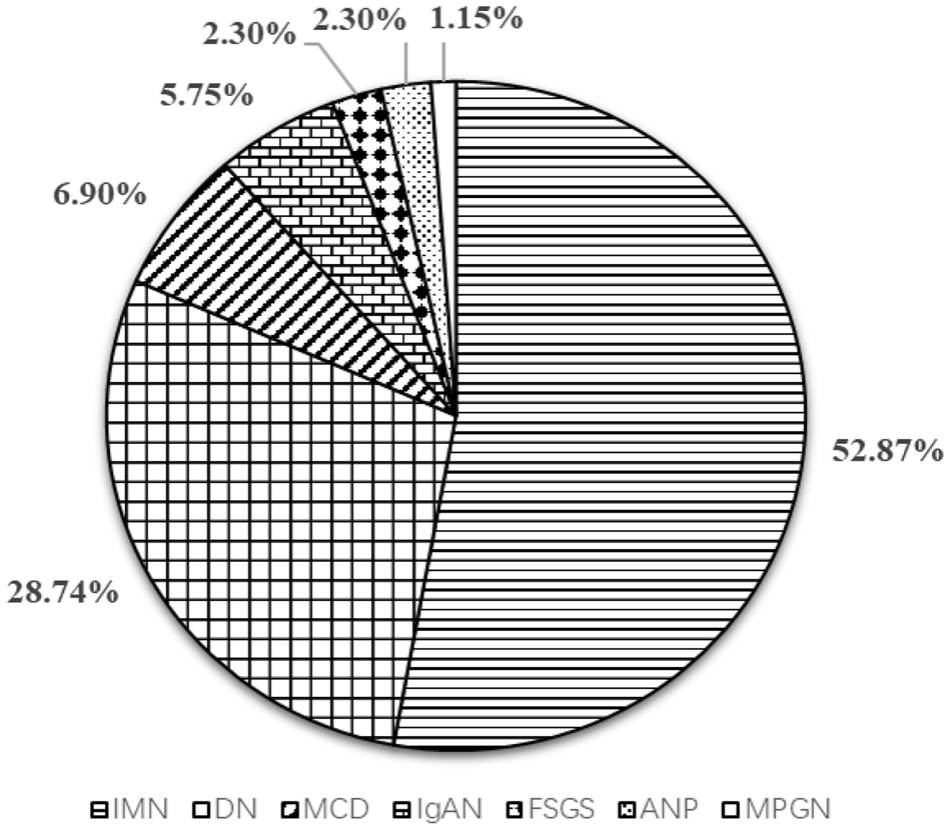
The distribution of pathological types in T2DM patients with proteinuria.

In this study, IMN (46 cases, 52.87%) was the most frequent glomerulopathy in T2DM patients with proteinuria, followed by DN (25 cases, 28.74%), minimal change disease (6 cases, 6.90%), IgA nephropathy (5 cases, 5.75%), focal segmental glomerulosclerosis (2 cases, 2.30%), amyloidosis nephropathy (2 cases, 2.30%), and membranoproliferative glomerulonephritis (1 case, 1.15%). As seen in Fig. 2. In addition, only three subjects (3.44%) had features of both DKD and NDKD.

**Figure 3:**
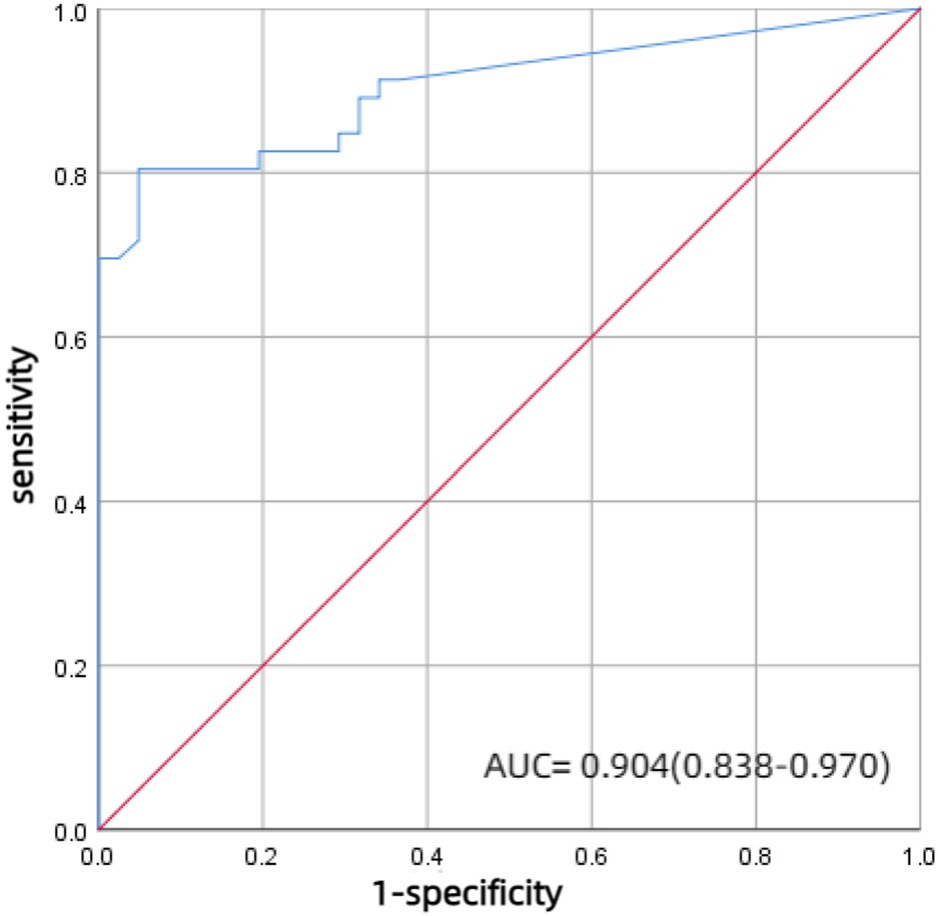
ROC curve of serum anti-PLA2R antibody in predicting IMN. (AUC = 0.904, sensitivity: 0.804%, specificity: 0.951%, *95%CI *0.838–0.970, *P* < 0.001).

The comparison of clinical baseline and biochemical characteristics among the three groups is shown in Table 1. Compared with the IMN group, the DN group had a longer duration of diabetes, more patients with DR, DPN, and hypertension, higher levels of SBP, ALB, BUN, Scr, HbA1c, and lower positive rate of anti-PLA2R antibody (*P* < 0.05). However, there was no significant difference in UTP between the two groups. Compared with other glomerulopathy groups, the DN group had a higher prevalence of DR and SBP, while the IMN group had a higher UTP level and anti-PLA2R antibody positive rate, but lower ALB, BUN, Scr, and eGFR (*P* < 0.05). In addition, there was no statistically significant difference in sex, age, BMI, family history of diabetes, DBP, UA, and FPG among the three groups (*P* > 0.05). Moreover, in this study population, the anti-PLA2R (AUC = 0.904, sensitivity: 0.804%, specificity:0.951%, *95%CI *0.838–0.970, *P* < 0.001) antibody has a high diagnostic accuracy for IMN (Fig. 3).

**Table 1:** Comparison of baselines characteristics among three groups.

Variable	IMN (n = 46)	DN (n = 25)	Others (n = 16)	*F*/*χ*^*2*^	*P*值
Male (n, %)	29(63.04%)	17(68.00%)	9(56.25%)	0.581	0.748
Age (Years)	54.00 (49.00, 64.00)	54.00 (46.50, 64.50)	57.00 (40.25, 65.00)	0.159	0.993
DM duration (Years)	1.00 (0.31, 4.00)	7.00 (3.00, 12.00)*	3.00 (0.75, 10.00)	15.922	< 0.001
BMI (kg/m^2^)	28.47 ± 3.74	26.69 ± 4.05	27.87 ± 4.93	1.559	0.216
DM family history (n, %)	5(10.87%)	5(20.00%)	3(18.75%)	1.541	0.521
DR (n, %)	8(17.39%)	20(80.00%)*	1(6.25%)^#^	35.042	< 0.001
DPN (n, %)	8(17.39%)	15(60.00%)*	6(37.50%)	13.386	0.001
Hypertension (n, %)	32(69.57%)	23(92.00%)*	15(93.75%)	6.796	0.026
SBP (mmHg)	146.41 ± 24.20	165.48 ± 30.72*	143.38 ± 20.38^#^	5.422	0.006
DBP (mmHg)	88.80 ± 13.09	92.48 ± 13.53	87.94 ± 14.05	0.780	0.462
ALB (g/L)	25.72 ± 6.00	30.92 ± 6.40*	30.35 ± 9.05*	5.929	0.004
BUN (mmol/L)	4.60 (3.73, 5.44)	6.92 (5.60, 9.94)*	6.15 (5.10, 7.32)*	22.520	< 0.001
Scr (umol/L)	66.85 (58.88, 75.41)	96.10 (78.13, 136.29)*	91.80 (65.67, 146.53)*	22.174	< 0.001
eGFR (ml/min·1.73m^2^)	103.43 (92.16, 117.57)	69.36 (49.03, 81.86)*	69.68 (44.46, 110.73)*	28.157	< 0.001
UA (umol/L)	352.42 (305.51, 440.05)	333.24 (293.87, 389.36)	356.77 (288.75, 470.37)	0.896	0.639
TG (mmol/L)	2.48 (1.55, 3.52)	1.60 (0.90, 2.24)*	1.91 (1.29, 3.95)	10.787	0.005
TC (mmol/L)	7.03 (6.00, 10.88)	6.76 (4.75, 7.39)*	6.03 (5.24, 8.11)	6.039	0.049
FPG (mmol/L)	6.06 (5.37, 7.36)	7.91 (5.06, 9.63)	6.86 (5.23, 7.85)	3.087	0.214
HbA1c (%)	6.70 (6.10, 7.25)	7.70 (6.50, 9.40)*	7.05 (6.50, 7.88)	7.071	0.029
UTP (g/24 h)	5.24 (4.11, 8.55)	6.16 (3.49, 7.09)	3.20 (2.51, 5.68)*	7.274	0.026
anti-PLA2R antibody ≥ 14(RU/ml)	37 (80.43)	1 (4.00)*	1 (6.25)*	50.058	< 0.001

*Compared with IMN group, *P* < 0.05, ^#^compared with DN group, *P* < 0.05.

To explore the risk factors for pathological diagnosis of IMN in T2DM patients with proteinuria, the indicators with statistical significance between IMN and DN groups in Table 1 were included in the multivariate logistic regression analysis. The results are shown in Tables 2 and 3. Shorter duration of diabetes (*OR* = 0.798, *P* = 0.030), higher eGFR levels (*OR* = 1.030, *P* = 0.024), and positive anti-PLA2R antibody (*OR* = 72.727, *P* < 0.001) are more likely to be diagnosed as IMN. Meanwhile, patients with higher SBP (*OR* = 1.041, *P* = 0.030), DR (*OR* = 50.234, *P* < 0.001), or negative anti-PLA2R antibody (*OR* = 0.008,* P* = 0.001) were more likely to be diagnosed as DN.

**Table 2:** Multivariate logistic regression analysis of IMN predictors.

Variable	*β*	*SE*	*Waldχ*^*2*^	*OR*	95%* CI*	*P*
DM duration	− 0.226	0.104	4.698	0.798	0.650–0.979	0.030
eGFR	0.029	0.013	5.115	1.030	1.004–1.056	0.024
anti-PLA2R antibody ≥ 14	4.287	0.960	19.939	72.727	11.080–477.372	< 0.001

**Table 3:** Multivariate logistic regression analysis of DN predictors.

Variable	*β*	*SE*	*Waldχ*^*2*^	*OR*	95%* CI*	*P*
DR	3.917	0.991	15.614	50.234	7.199–350.518	< 0.001
SBP	0.040	0.018	4.691	1.041	1.004–1.079	0.030
anti-PLA2R antibody ≥ 14	− 4.830	1.429	11.424	0.008	0.000–0.131	0.001

## Discussion

The prevalence of T2DM has gradually increased in recent years. DN has surpassed primary glomerular disease as the main cause of chronic kidney disease (CKD) and end-stage kidney disease (ESKD)^23, 24^. However, many diabetic patients with proteinuria are classified as NDKD in pathology. Kidney biopsy is the gold standard for pathological diagnosis up to now. But in some undeveloped areas, there are limited conditions to perform kidney biopsy. How to identify the pathological types by non-invasive examination for such patients is becoming an urgent problem.

In our study, 71.26% of patients with T2DM combined with proteinuria were NDKD, among which IMN accounted for 52.87%. The IMN group had an anti-PLA2R antibody positivity rate as high as 80.43%. It suggested that the pathological type of these patients was more likely to be NDKD or DN combined with NDKD when the anti-PLA2R antibody was positive. This is consistent with previous studies. For IMN, anti-PLA2R antibody has higher diagnostic accuracy (sensitivity: 0.804%, specificity: 0.951%)^7, 25,26^.

NDKD may be considered when diabetic patients have proteinuria but a short duration of disease. Our study showed the same results as the others^27–29^. In addition, DR had a close correlation with the presence of DN, which means that patients without DR were more likely to be diagnosed with NDKD^10^. However, there are still some severe DR patients without DN^30^. Nevertheless, this does not deny the diagnostic value of DR. In this study, the frequency of hypertension in the DN group was higher than in the other two groups, which was consistent with the results of Dong et al^8^. Hypertension not only accelerates the progression of DN but is also an important risk factor for DN. Moreover, it is associated with macrovascular and microvascular complications in T2DM patients^31^. Overall, the kidney prognosis of patients with NDKD is better than that of patients with DN^32^. In our study, patients with higher eGFR levels are more likely to be diagnosed with IMN. However, this prediction is unstable and needs long-term follow-up to prove it. Compared with NDKD patients, DN patients are more prone to suffer from anemia^33^, monitoring of hemoglobin levels may help to identify DN or NDKD^34^. There is no significant difference in FPG between the DN group and the other two groups, but the level of HbA1c is higher than those two groups, which is different from the results of Liu et al^35^.

T2DM patients with proteinuria who have short DM duration, higher eGFR levels, and positive anti-PLA2R antibody tend to be diagnosed as NDKD, and its pathological type is IMN. By contrast, DR patients with negative anti-PLA2R antibody were more likely to be diagnosed as DN. Nonetheless, for the latter, kidney biopsy is still recommended. Anti-PLA2R antibody play an important role in distinguishing DN and NDKD, but they still can not replace kidney biopsy as a diagnostic criterion.

There are several limitations to this study. First, nonalbuminuric diabetic kidney disease (NADKD) was not included in this study. This clinical type of DN usually has a short course of diabetes, and it is rarely complicated with DR^36^. Additionally, the underlying pathology of NADKD might be different from that of the classic phenotype of DN. However, the recruitment of such patients may have a subtle impact on the current outcomes, as the number of them is small. Additionally, although anti-PLA2R antibody has shown high diagnostic accuracy due to the small number of samples in this study, large-scale studies are still needed in the future to verify the conclusion.

## Data Availability

The data that support the findings of this study are available from the corresponding author upon reasonable request.
